# Analytical Evaluation of Signal-to-Noise Ratios for Avalanche- and Single-Photon Avalanche Diodes

**DOI:** 10.3390/s21082887

**Published:** 2021-04-20

**Authors:** Andre Buchner, Stefan Hadrath, Roman Burkard, Florian M. Kolb, Jennifer Ruskowski, Manuel Ligges, Anton Grabmaier

**Affiliations:** 1 Fraunhofer Institute for Microelectronic Circuits and Systems, Finkenstr. 61, 47057 Duisburg, Germany; jennifer.ruskowski@ims.fraunhofer.de (J.R.); manuel.ligges@ims.fraunhofer.de (M.L.); anton.grabmaier@ims.fraunhofer.de (A.G.); 2 OSRAM GmbH, Nonnendammallee 44, 13629 Berlin, Germany; stefan.hadrath@osram.com (S.H.); F.Kolb@osram.com (F.M.K.); 3 Department of Electronic Components and Circuits, University of Duisburg-Essen, Bismarckstr. 81, 47057 Duisburg, Germany; roman.burkard@uni-due.de

**Keywords:** SPAD, APD, LiDAR, SNR, time-of-flight, multi-event

## Abstract

Performance of systems for optical detection depends on the choice of the right detector for the right application. Designers of optical systems for ranging applications can choose from a variety of highly sensitive photodetectors, of which the two most prominent ones are linear mode avalanche photodiodes (LM-APDs or APDs) and Geiger-mode APDs or single-photon avalanche diodes (SPADs). Both achieve high responsivity and fast optical response, while maintaining low noise characteristics, which is crucial in low-light applications such as fluorescence lifetime measurements or high intensity measurements, for example, Light Detection and Ranging (LiDAR), in outdoor scenarios. The signal-to-noise ratio (SNR) of detectors is used as an analytical, scenario-dependent tool to simplify detector choice for optical system designers depending on technologically achievable photodiode parameters. In this article, analytical methods are used to obtain a universal SNR comparison of APDs and SPADs for the first time. Different signal and ambient light power levels are evaluated. The low noise characteristic of a typical SPAD leads to high SNR in scenarios with overall low signal power, but high background illumination can saturate the detector. LM-APDs achieve higher SNR in systems with higher signal and noise power but compromise signals with low power because of the noise characteristic of the diode and its readout electronics. Besides pure differentiation of signal levels without time information, ranging performance in LiDAR with time-dependent signals is discussed for a reference distance of 100 m. This evaluation should support LiDAR system designers in choosing a matching photodiode and allows for further discussion regarding future technological development and multi pixel detector designs in a common framework.

## 1. Introduction

Signal-to-noise ratio (SNR) is a widely used metric for the ability of a photodetector to distinguish between an incident signal and its absence. While SNR is frequently invoked to classify detector sensing capabilities, discourse is often limited to qualitative expressions like ‘high’ and ‘low’ SNR without further defining the analytical origins of SNR for different detectors or discussing specific, quantitative examples.

Various research groups have compared the performance of APD- and SPAD-based optical detection systems and also derived SNR definitions for both APDs and SPADs. The authors of [1] derive an SNR definition for SPADs in single- or multi-event [2,3] acquisition mode which is the basis for this work. An extension of this definition is given by [4] representing afterpulsing effects in gated SPADs, which are assumed to be negligible in this work through the use of a sufficiently long dead time.

APDs and their readout circuitry on the other hand are a comparatively mature technology whose noise sources are well-understood and mathematically described. The SNR depending on the chosen readout electronics is extracted from standard literature [5,6].

Comparisons of APD and SPAD performance for different applications are also reported in literature. A recent publication which conducts a similar comparison to this one is [7]. The authors derive their results using a Monte-Carlo based photonic simulator. The difference between their results and the results detailed in this publication is that their simulations are based on a SPAD system which may accumulate multiple measurements while the compared APD system has to create its result from a single measurement and no analytical expressions of the SNR are given. By contrast, we decided to focus on a scenario in which measurement accumulation for APD and SPADs are kept at the same level, even if accumulation can be considered necessary for SPAD detectors and optional for APD detectors. The author of [8] evaluates the respective diode’s performance in an optical wireless communications scenario. The comparison is conducted in a low ambient light scenario and the saturating behavior of the SPAD detector is not taken into account. Reference [9] compares SPAD and APDs directly for their application in visible light communications but uses an approximation for the SPAD performance which is based on its photon-counting performance and does not consider the characteristics of time-correlated acquisition. The authors of [10] compare different options for flash LiDAR detectors with focus on GaAs-based detectors.

This work advances the state-of-the-art in that it considers multi-event and asynchronous acquisition of photoelectron-generated events for SPADs, not limited to low-flux scenarios. The authors are not aware of any publication which considers the saturating behavior of SPAD SNR in ambient light scenarios and compares them to the respective APD behavior based on SNR values, including the readout circuitry, of commercially available diodes in ranging applications. Recommendations are given which can provide a basis for the design of future electrooptical systems, and equations are presented which will allow the reader to scale the considerations for their own design efforts.

Section 2 deals with the properties of optical signal acquisition using APD and SPAD receivers. Effects of their noise behavior in relation to their respective readout electronics are discussed briefly and mitigation strategies are sketched.

In Section 3, the detector types are analytically compared by evaluating the equations over a range of input powers. The advantages of SPADs in low-light and short to mid-range ranging applications are shown. Besides a performance estimation based on exemplary parameters from commercially available photodiodes, the impact of future parameter improvements is discussed. The advantageous properties of APDs in systems with overall high signal levels are displayed.

Section 4 discusses the findings and provides an outlook for possible technological perspectives and a summary.

## 2. Model and Method

The random nature of photon arrivals leads to a statistical detection process. In general, the values of noise and signal decide how probable it is to detect a signal over present noise. In the following, mathematical models of APDs and SPADs describing the stochastic behavior are presented. 

### 2.1. Time-Dependent Signals from APDs

A linear-mode APD operates under a reverse-bias voltage that is sufficient to enable avalanche multiplication. In contrast to a SPAD, this reverse-bias voltage is below the breakdown voltage of the APD and the multiplication results in an internal current gain, which is proportional to the incoming optical signal $P_{S}$ and noise power $P_{N}$. The conversion and multiplication into a measurable photocurrent $I_{S}$ are influenced by several noise sources. In the following, the basic principle of avalanche multiplication process and noise sources of an APD are briefly summarized.

The process of the avalanche multiplication is started by an optically or thermally generated electron in the depletion region of the p-n junction. The electric field, caused by the applied reverse-bias voltage, accelerates this electron. If a sufficient reverse-bias voltage is applied, the electron gains enough kinetic energy to create new electron-hole pairs. These newly created electron-hole pairs are again accelerated by the electric field and may create further electron-hole pairs, resulting in an overall avalanche process summarized by the multiplication factor M [5].

#### 2.1.1. APD Noise Sources

Besides the dependency of the multiplication factor M on the biasing voltage of the APD, this avalanche multiplication process is an inherently random process and introduces a noise source known as avalanche noise, as not every generated electron-hole pair experiences the same multiplication. Avalanche noise is described using the noise factor F, given in Equation (1), and depends on the ionization coefficients $\alpha_{p}$ and $\alpha_{n}$ of the carriers (holes and electrons respectively) and the multiplication factor M [5]. For silicon APDs, the ratio of the ionization coefficients αpαn is low, and typical noise factor values range from 2 to 3 [6].

(1) $$F=\frac{\left\langle M^{2}\right\rangle }{M}=M\left(\frac{\alpha_{p}}{\alpha_{n}}\right)+\left(2-\frac{1}{M}\right)\left(1-\frac{\alpha_{p}}{\alpha_{n}}\right).$$

The process of the avalanche multiplication may be started by the presence of a thermally generated electron. With other non-optical noise sources this is combined into a dark current $I_{d}$. A distinction in the dark current is made between an unmultiplied (surface) $I_{\mathrm{ds}}$ and a multiplied (bulk) $I_{\mathrm{db}}$ current term, which is expressed as

(2) $$I_{d}=I_{\mathrm{ds}}+I_{\mathrm{db}}M.$$

The surface dark current $I_{\mathrm{ds}}$ contributes shot noise only, whereas the bulk dark current $I_{\mathrm{db}}$ undergoes avalanche multiplication and therefore contributes excess noise. The shot noise current density $\left\langle I_{d}\right\rangle$ contribution of the total dark current is

(3) $$\langle I_{d}\rangle =\sqrt{2e\left(I_{\mathrm{ds}}+I_{\mathrm{db}}M^{2}F\right)},$$

where e denotes the elementary charge. In the following, we distinguish between an application-specific desired (time-dependent) signal current $I_{S}$ and an underlying (constant) background signal $I_{B}$ caused by solar background in laser ranging applications, for example. Summarizing the current contributions from the aforementioned sources, an expression for the output current I  of the APD can be found to be

(4) $$I=I_{\mathrm{ds}}+\left(I_{S}+I_{B}+I_{\mathrm{db}}\right)M$$

and the shot noise current density I of this current I is

(5) $$\langle I\rangle =\sqrt{2e\left(I_{\mathrm{ds}}+\left(I_{S}+I_{B}+I_{\mathrm{db}}\right)M^{2}F\right)}.$$

#### 2.1.2. APD Noise Sources Including Readout Circuitry

**Figure 1:**
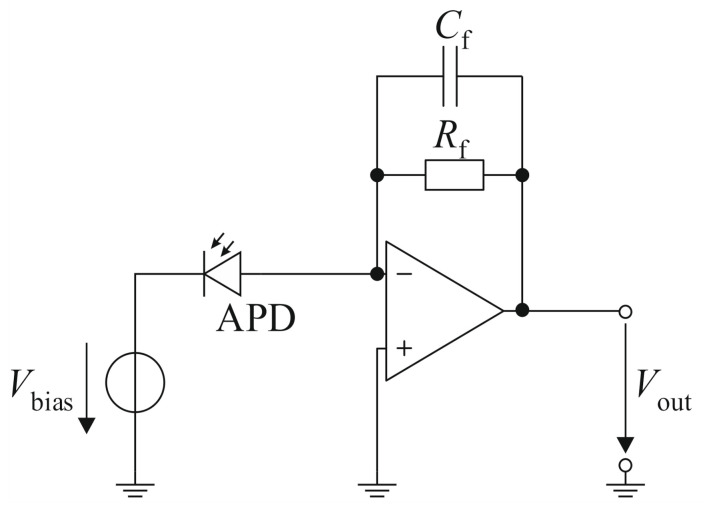
APD with biasing voltage and transimpedance amplifier.

To further describe the noise properties of an APD, a simple example of a read-out circuit for an APD needs to be considered. This read-out circuit is shown in Figure 1 and consists of a voltage source, which is used to supply the reverse-bias voltage $V_{\mathrm{Bias}}$ of the APD, and a transimpedance amplifier, which converts and further amplifies the photocurrent into a proportional output voltage $V_{\mathrm{out}}$. In first-order approximations, the output voltage caused by the signal power is

(6) $$V_{\mathrm{out}}=I_{S}\cdot M\cdot R_{f}=R^{\prime }\cdot M\cdot P_{S}\cdot R_{f}=R\cdot P_{S}\cdot R_{f},$$

where R and R’ are the responsitivity of the APD with and without the multiplication factor, respectively, and $P_{S}$ is the signal power impinging on the sensor. Further typical assumptions concerning the amplifier are a 3 dB bandwidth $B_{N}$ that matches the rise time of the photocurrent pulse, a linear response and no stray capacitances. Furthermore, the noise contribution of the amplifier is described by adding a noise current density $\left\langle I_{\mathrm{amp}}\right\rangle$ to its output.

(7) $$\langle V_{\mathrm{amp}}\rangle =\langle I_{\mathrm{amp}}\rangle R_{f}$$

With the respective amplification voltage $V_{\mathrm{Amp}}$ which is derived by multiplication with the bandwidth $B_{N}$ following Equation (8)

(8) $$V_{\mathrm{amp}}=\langle V_{\mathrm{amp}}\rangle \sqrt{B_{N}}.$$

The resistor $R_{f}$ sets the gain of the transimpedance amplifier and contributes a Johnson noise current of

(9) $$\langle I_{\mathrm{Rf}}\rangle =\frac{4k_{B}T}{R_{f}},$$

where T is the temperature and $k_{B}$ is the Boltzmann constant. The capacitor $C_{f}$ is used as a phase compensation and reduces the gain peaking of the read-out circuit at higher frequencies [11].

Adding the aforementioned noise currents in quadrature and transforming these into a voltage yields the total noise voltage $\left\langle V_{\mathrm{out}}\right\rangle$ at the output of the amplifier of

(10) $$\langle V_{\mathrm{out}}\rangle =\sqrt{2eB_{N}\left(I_{\mathrm{ds}}+\left(I_{S}+I_{B}+I_{\mathrm{db}}\right)M^{2}F\right)R_{f}^{2}+B_{N}\left(4k_{B}TR_{f}+\langle V_{\mathrm{amp}}^{2}\rangle \right)},$$

where the current $I_{B}$ is the current caused by background radiation and the bandwidth $B_{N}$ is the noise bandwidth of the amplifier. In contrast to the signal bandwidth B, the noise bandwidth $B_{N}$ is defined as the frequency at which the gain of the amplifier becomes 0 dB. Only in the limiting case, where the frequency response of the amplifier is assumed to be an ideal low-pass filter, both bandwidths are equal.

#### 2.1.3. APD SNR

The ratio of the signal output voltage and the total noise voltage is defined as the SNR of the APD and the read-out circuit and is given in Equation (11) [12]

(11) $${\mathrm{SNR}}_{\mathrm{APD}}=\frac{I_{S}R_{f}}{\sqrt{2eB_{N}\left(I_{\mathrm{ds}}+\left(I_{S}+I_{B}+I_{\mathrm{db}}\right)M^{2}F\right)R_{f}^{2})+4k_{B}B_{N}TR_{f}+B_{N}\langle V_{\mathrm{amp}}^{2}\rangle }}$$

In terms of optical power and assuming a high multiplication factor M and N individual signal acquisitions, this may be simplified and expressed as

(12) $${\mathrm{SNR}}_{\mathrm{APD}}=\sqrt{\frac{NR^{\prime 2}P_{S}^{2}}{2eB_{N}\left(R^{\prime }P_{S}+R^{\prime }P_{B}+I_{\mathrm{db}}\right)F+\frac{1}{M^{2}}B_{N}\left(\frac{4k_{B}T}{R_{f}}+\frac{\left\langle V_{\mathrm{amp}}^{2}\right\rangle }{R_{f}^{2}}\right)}}.$$

When placing multiple pixels in an illuminated area, each of them will only have a fraction of incident photons impinging on its respective surface. Furthermore, not all of the pixel area is optically active which can be expressed by introducing a fill-factor $\eta_{\mathrm{FF}}$ as the ratio of optically active to total area. Accounting for the power reduction of both leads to the expression in (13)

(13) $$P_{S}=\frac{\eta_{\mathrm{FF}}P_{S}^{\prime }}{N_{\mathrm{APD}}}.$$

When accounting for finite fill-factor and summing the signals of $N_{\mathrm{APD}}$ different detectors, Equation (12) extends to (14)

(14) $${\mathrm{SNR}}_{\mathrm{APD}}=\sqrt{\frac{NN_{\mathrm{APD}}\eta_{\mathrm{FF}}^{2}\;R^{\prime 2}P_{S}^{\prime }{}^{2}}{2eB_{N}\left(\frac{R\prime \eta_{\mathrm{FF}}P_{S}}{N_{\mathrm{APD}}}+\frac{R\prime \eta_{\mathrm{FF}}P_{B}}{N_{\mathrm{APD}}}+I_{\mathrm{db}}\right)F+\frac{1}{M^{2}}B_{N}\left(\frac{4k_{B}T}{R_{f}}+\frac{\left\langle V_{\mathrm{amp}}^{2}\right\rangle }{R_{f}^{2}}\right)}}.$$

### 2.2. Single-Photon Counting Using SPADs

A SPAD is a photodiode which is brought into a photosensitive metastable state by biasing it above its breakdown voltage. The high voltage drop-off over the active region of the SPADs accelerates photo-generated carriers high enough to generate a large number of additional carriers and trigger a macroscopic current, causing the device to leave its metastable state. This makes the SPAD sensitive for single incident photons. In contrast to the APD, this current is not proportional to the initial photocurrent but quickly reaches saturation through an amplification which approaches infinity.

For measuring time-dependent signals, this change in current can be detected via a time-to-digital converter (TDC). The output pulse of the SPAD undergoes a pulse-shaping circuit and triggers the TDCs input. There are evaluation circuits which solely count the number of occurring events while being active. Other implementations are capable of resolving the relative time of arrival of the event and enable time-correlated single photon counting (TCSPC) applications with higher accuracy requirements.

Similar to the APD, incident photons can miss the optically active area. This leads to the definition of a fill-factor $\eta_{\mathrm{FF}}$, which is simply a geometrical ratio between optically active and inactive area. Photons hitting the optically active area have to generate a photoelectron which is subsequently accelerated to cause an avalanche breakdown. Those effects also happen with a finite efficiency, which is summarized in the photon detection probability (PDP) $\eta_{\mathrm{PDP}}$.

If the breakdown current is sustained, the diode becomes insensitive to further impinging photons, and the associated power dissipation could lead to the thermal destruction of the photodiode. Thus, it must be reset to the previous state. To reset, the biasing voltage must be reduced far enough below the breakdown voltage to stop the current flow and then increased again.

The time that it takes to get the SPAD ready for the next detection is called dead time. During dead time, the SPAD is not sensitive for additional incident photons. The act of resetting is called quenching. Quenching circuits can be subdivided into different categories. Passive quenching can be realized using a resistor connected in series to the SPAD to lower the voltage over the diode under the breakdown voltage after an avalanche occurs. Passive quenching circuits are realized with a high impedance termination and their recovery times are on the order of microseconds [13].

**Figure 2:**
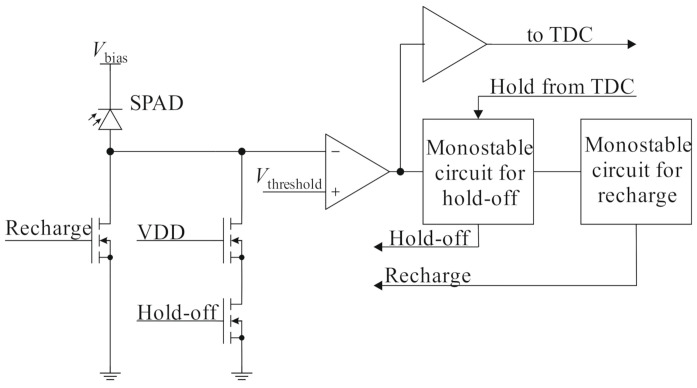
SPAD with a combined active/passive quenching circuit from [16].

Today’s active quenching circuits achieve dead times in the order of 20 ns [14] while sufficiently suppressing afterpulsing, with some systems exhibiting dead times down to a few nanoseconds [15]. This time is a tradeoff between the maximum number of potential photon acquisitions per measurement and the necessity to deactivate the SPAD long enough to suppress afterpulsing effects caused by trapped carriers in interband states with exponentially decaying lifetime [13]. Especially short dead times can be achieved using combinations of the fast voltage drop-off of passive quenching and active elements to keep dead times low. An exemplary circuit with a low dead time is shown in Figure 2.

#### 2.2.1. SPAD Noise Sources

An avalanche can be triggered not only by incident photons but also via carriers that were generated thermally or through band-to-band tunneling [17]. Those carriers are accelerated just like the electron hole pairs which were generated via the inner photoelectric effect. Since the avalanche breakdown is triggered in the absence of light, the corresponding noise level is referred to as dark count rate (DCR) $r_{\mathrm{DCR}}$. As a single avalanche can either stem from noise or signal and it is impossible to distinguish them without the use of additional information or statistics on multiple accumulated measurements, the DCR must be kept low.

An increase in bias voltage increases both the dark count rate and the probability for triggering an avalanche after an electron-hole pair has been generated. Therefore, a tradeoff between SPAD sensitivity and DCR must be found. Furthermore, higher temperatures also lead to an increase in DCR [18] due to thermal activation of carriers. 

In high ambient light level scenarios, the impact of DCR is less drastic because the ambient light levels usually produce an intrinsic shot noise level which is orders of magnitude higher. 

The sudden increase in current caused by a triggered avalanche produces an electromagnetic field which can in turn trigger other detectors in the vicinity. This effect is called crosstalk. It is assumed that through careful design, for example, using isolating trenches between detectors and a sufficiently large pixel pitch, the amount of crosstalk is negligible for the consideration of the SNR.

When an avalanche breakdown has occurred, and the SPAD is quenched, carriers can remain in intermediate band states, with occupation probability decreasing exponentially due to their lifetime. When $V_{\mathrm{Bias}}$ is increased again too soon, those carriers can lead to another breakdown, which is correlated to the first one and is called an afterpulse. Afterpulsing is assumed to be negligible with probabilities below <1%. Measurement schemes based on first photon acquisition do not suffer from afterpulsing effects because after its first detection the SPAD is being disabled and is thus unable to detect further avalanches. In that regard, they are similar to systems with an infinitely high dead time.

#### 2.2.2. Photon Detection Statistics and Operation Modes

Optical detectors aim to detect the presence or absence of a signal photon stream. While the APD noise is expressed in terms of occurring noise currents, a description based on the event rate is more common with SPAD detectors. The number of incident photons N, which stem from a photon source, for example, lasers or the sun, arriving in any given time interval T  can be modelled using a Poisson point process with rate parameter r. The value of r is proportional to the incident optical power $P\left(t\right)$ of energy $W_{\mathrm{Ph}}=\frac{hc}{\lambda }\;$ on a detector with photon detection efficiency $\eta_{\mathrm{PDE}}$ [19]. For constant signal $P_{S}$ and background light power $P_{B}$ this results in the photon electron rates

(15) $$r_{L}=\frac{\eta_{\mathrm{PDE}}}{W_{\mathrm{Ph}}}P_{S}$$

and

(16) $$r_{B}=\frac{\eta_{\mathrm{PDE}}}{W_{\mathrm{Ph}}}P_{B}.$$

Photon detection efficiency $\eta_{\mathrm{PDE}}$ combines signal loss through the geometrical fill-factor $\eta_{\mathrm{FF}}$ and the finite probability of conversion from incident photons to triggered avalanche $\eta_{\mathrm{PDP}}$.

(17) $$\eta_{\mathrm{PDE}}=\eta_{\mathrm{FF}}\eta_{\mathrm{PDP}}.$$

The expected number of arrivals m in a time interval T produced by light sources with Poissonian statistics and constant intensity with flux $r_{B}$ for k occurring detections can be described using the distribution function in Equation (19).

(18) $$m=\int_{0}^{T}r\left(t\right)\;\mathrm{dt}=\frac{\eta_{\mathrm{PDE}}}{W_{\mathrm{Ph}}}\int_{0}^{T}P\left(t\right)\;\mathrm{dt}$$

(19) $$p\left(t\right)=\frac{m^{k}}{k!}\exp\left(-m\right)$$

A property of the Poisson process is that its standard deviation σ of counted photons is linked to the mean value of arrivals

(20) $$\sigma =\sqrt{m}.$$

The most common acquisition mode for SPADs which is used in a variety of applications is single-photon acquisition. The measurement is stopped after the first avalanche breakdown. This avoids the effects of the dead time on signal acquisition but introduces signal-dependent saturation into the system. The probability density function of one or more detections with a constant rate r follows (21).

(21) $$P_{A}\left(r,t\right)=r\exp\left(-m\right)$$

Pushing dead times into the nanosecond regime enables a different acquisition mode for SPADs. In combination with so-called multi-hit TDCs which are capable of acquiring multiple avalanche timestamps in rapid succession, saturation effects through noise photons can be partially mitigated.

Assuming measurement cycles of finite length $t_{\mathrm{ToF}}$ and finite dead time $t_{D}$, the maximum number of photons $N_{\mathrm{Ph}}$ is limited. The longer of the two dead times of TDC $t_{D,\mathrm{TDC}}$ and SPAD $t_{D,\mathrm{SPAD}}$ has to be considered here because both limit the capability of acquiring more photons in the same way

(22) $$N_{\mathrm{Ph}}=\lceil \frac{t_{\mathrm{ToF}}}{\max\left(t_{D,\mathrm{TDC}},\;t_{D,\mathrm{SPAD}}\right)}\rceil .$$

The probability density for this multi-event acquisition is valid when the detector is still unblocked because fewer than the maximum number of photon detections $N_{\mathrm{Ph}}$ have occurred previously [1]. Subsequent events are delayed by a dead time between the detection of these events, resulting in the total PDF $P_{A,M}$ in (23)

(23) $$P_{A,M}\left(r_{B},t\right)=\overset{N_{\mathrm{Ph}}}{\underset{k=1}{\sum }}\frac{r_{B}^{k}{\left(t-\left(k-1\right)t_{D}\right)}^{k-1}}{\left(k-1\right)!}\exp\left(-r_{B}\left(t-\left(k-1\right)t_{D}\right)\right).$$

For $t_{D}=0$ this equation simplifies to an Erlang distribution. $N_{\mathrm{Ph}}$ is the maximum number of photons assuming an infinitely high photon flux. This is an idealization because the next arrival is generally not instantaneous, and a photon inter-arrival time has to pass after each detection before the next avalanche is triggered. When setting $N_{\mathrm{Ph}}=1$, the single photon detection PDF (21) follows from Equation (23).

#### 2.2.3. SPAD Histogram SNR for Time-Dependent Signals

A histogram is formed by accumulating time-resolved measurements with a certain resolve time interval $t_{\mathrm{Bin}}$. The flux of a rectangular pulse with infinitely steep slopes in a constant background level $r_{B}$ is realized by a piecewise constant function, where Φ denotes the Heaviside function (24)

(24) $$r\left(t\right)=\Phi \left(t\right)\cdot r_{B}+(\Phi \left(t-t_{\mathrm{ToF}}\right)-\Phi \left(t-(t_{\mathrm{ToF}}+t_{P})\right)\cdot r_{L}.$$

A rectangular pulse constitutes an idealization of laser pulse form. It can be regarded as the upper limit of reachable SNR for a pulse of arbitrary form with pulse peak power corresponding to $r_{L}$. The SNR in this case is limited by the saturation through background light photons up to the time $t_{\mathrm{ToF}}$ and the mean number of detections during a pulse time $t_{P}$. The SNR follows [20]

(25) $${\mathrm{SNR}}_{\mathrm{Single}}=\sqrt{Nt_{P}\frac{P_{A}}{r_{B}}}\frac{r_{L}}{\sqrt{r_{L}+r_{B}}}.$$

For a shorter acquisition time $t_{\mathrm{Bin}}$ than $t_{P}$ this value is limited to the expression in (26) because of the lower mean values in a smaller time interval

(26) $${\mathrm{SNR}}_{\mathrm{Single}}=\sqrt{Nt_{\mathrm{Bin}}\frac{P_{A}}{r_{B}}}\;\frac{r_{L}}{\sqrt{r_{L}+r_{B}}}.$$

In contrast to the definition of SNR for the APD, this SNR definition for SPADs is only defined for a single time interval $t_{\mathrm{Bin}}$. If that time interval is matched to the pulse duration, the total pulse energy contributes to the ranging result.

In case of multi-event detection, the SNR can benefit from the reduced saturation level $P_{A,M}$ analogous to Equation (23)

(27) $${\mathrm{SNR}}_{\mathrm{Multi},\mathrm{Event}}=\sqrt{Nt_{\mathrm{Bin}}\frac{P_{A,M}}{r_{B}}}\frac{r_{L}}{\sqrt{r_{L}+r_{B}}}\;.$$

The usually smaller footprint of SPADs in comparison to APDs makes it possible to fit more than one SPAD in the same area that an APD would occupy. Assuming homogeneous illumination and the integration of each SPAD with an active quenching circuit and its own TDC, the received input power is split up equally between the $N_{\mathrm{SPAD}}$ detectors. A common histogram can profit from the higher possible number of timestamps used for measurement evaluation. DCR values stemming from single SPADs are more pronounced because only incident rates are reduced, but DCR per detector stays constant. In reality, smaller detectors exhibit lower DCR and thus further increase performance. The reduced rates for the background $r_{B}^{\prime }$ and the laser $r_{L}^{\prime }$ may be expressed according to (28) and (29).

(28) $$r_{B}^{\prime }=\frac{1}{N_{\mathrm{SPAD}}}r_{B}+r_{\mathrm{DCR}}$$

(29) $$r_{L}^{\prime }=\frac{1}{N_{\mathrm{SPAD}}}r_{L}$$

This yields Equation (30) when assuming a common histogram and homogeneous illumination.

(30) $$\begin{array}{cc} {\mathrm{SNR}}_{\mathrm{Multi},\mathrm{Event},\mathrm{Pixel}} & =\sqrt{NN_{\mathrm{SPAD}}t_{\mathrm{Bin}}\frac{1}{r_{B}^{\prime }}\;P_{A,M}\left(r_{B}\prime ,\;t_{\mathrm{ToF}}\right)\;}\frac{r_{L}^{\prime }}{\sqrt{r_{L}^{\prime }+r_{B}^{\prime }}}\\ & =\sqrt{Nt_{\mathrm{Bin}}\frac{1}{r_{B}^{\prime }}\;P_{A,M}\left(r_{B}\prime ,\;t_{\mathrm{ToF}}\right)\;}\frac{r_{L}}{\sqrt{r_{L}+r_{B}+N_{\mathrm{SPAD}}r_{\mathrm{DCR}}}} \end{array}.\;$$

Provided that a SPAD evaluation circuit is capable of detecting an unlimited number of events through either memory which can hold a sufficient number of timestamps or a readout scheme which is capable of transporting the data out of the sensor before the next event, $N_{\mathrm{Ph}}$ is solely limited by dead time and ranging distance and takes its maximum value according to (22).

In systems with very short dead time, the saturation-dependent factor approaches unity and the system’s performance is limited solely by the acquisition time $t_{\mathrm{Bin}}$ and photon shot noise.

(31) $${\mathrm{SNR}}_{\mathrm{Linear}}=\sqrt{Nt_{\mathrm{Bin}}}\frac{r_{L}}{\sqrt{r_{L}+r_{B}}}\;$$

This is the corner case of a linear detector. It is in practice unreachable but nevertheless constitutes a limiting case. When time-gating schemes or region-of-interest scanning are possible, one can also avoid these saturation effects and conduct measurements approaching the highest possible SNR.

## 3. Analytic Comparison of APD and SPAD Performances

The following section deals with an exemplary comparison of APD- and SPAD-based detectors for a laser-based ranging (LiDAR) scenario. Although the general idea for conducting such a comparison based on available laser and background power is here presented, it must be adapted to the technology currently available on the market and for the reader. A hypothetical target is placed at a reference distance of 100 m corresponding to $t_{\mathrm{ToF}}=667\;\mathrm{ns}$ and illuminated by a laser pulse with a rectangular temporal pulse profile of duration $t_{P}=8\;\mathrm{ns}$, wavelength of λ=905 nm and varying optical power. Previously introduced equations are used to calculate and plot values for SNR for each diode and all illumination scenarios.

### 3.1. General Comparison of Signal-to-Noise Ratios

**Figure 3:**
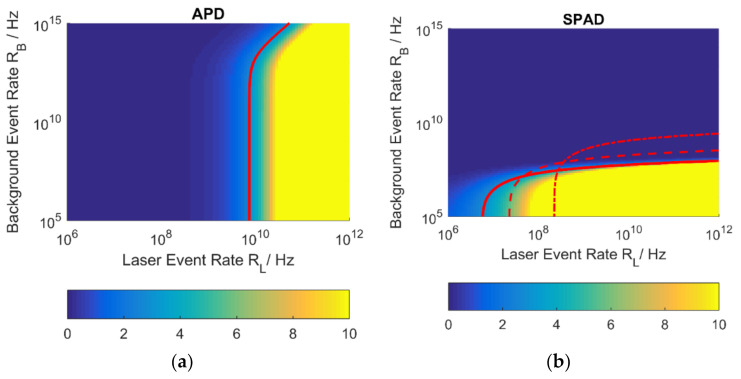
SNR for a single pixel SPAD and APD detector for a distance of 100 m and N=1000 accumulations. (**a**) SNR values for APD [21] (Table 1). (**b**) Achievable SNR for a SPAD detector in single photon mode with parameters according to Table 2 and $N_{\mathrm{SPAD}}=1$. SNR=3 drawn as red contour for $\eta_{\mathrm{PDE}}=0.2$ (solid line), $\eta_{\mathrm{PDE}}=0.05$ (dashed line) and $\eta_{\mathrm{PDE}}=0.005$ (dot-dashed line).

Values of SNR for APD [21] and a single-event SPAD according to Equations (12) and (25) and Table 1 and Table 2*,* respectively, are plotted in Figure 3. The common parameters for the LiDAR scenario used are again summarized in Table 3.

A wavelength in the near infrared was chosen because the human eye is not sensitive for this regime and it is a common wavelength for silicon-based detectors. The diode parameters from [7] are listed as reference values because they are the basis of the comparison conducted in [7] and facilitates a direct comparison to the results from this work. It is not considered further because it is optimized for a wavelength different than that of the APDs used in the comparisons.

The same detector area, fill-factor and optics for SPAD and APD are assumed. Values of SNR higher than 10 are expected to be easily evaluable providing high probability of detection and low false alarm probability and are thus plotted in the same color. The absolute value of required SNR for a given probability of detection and false alarm probability depends on the algorithm which is used to evaluate the measurement signal [22,23,24,25]. Alternatively, interframe, interpixel information, more processing intensive evaluation schemes or decision threshold choice can enable more successful detections [26]. In the literature, values between 5 and 10 are reported to constitute a desirable operating window [27], so a contour line is drawn at an SNR value of 3 as an evaluable, albeit challenging, scenario.

For the comparison plots, event rates $R_{B}$ and $R_{L}$ are defined which represent the total number of incident photons on a detector

(32) $$R_{L}=\frac{P_{S}}{W_{\mathrm{Ph}}}$$

and

(33) $$R_{B}=\frac{P_{B}}{W_{\mathrm{Ph}}}.$$

The APDs used in the comparison are evaluated based on a few common parameters. A transimpedance amplifier with $B_{N}=50\;\mathrm{MHz}$ and amplification voltage density $\langle V_{\mathrm{amp}}\rangle =$ 1.414 × 10^−7^ $\;\frac{V}{\sqrt{\mathrm{Hz}}}$ terminated with a resistance of $R_{f}=1000\;\Omega$ at an effective input noise temperature T=300 K is assumed for all following comparisons.

In this particular scenario, the first-photon detection capability of SPADs results in comparatively small incident background rates that can lead to the saturation of the detector at long ranges. This effect is especially dominant here because of the working distance of 100 m. Smaller maximum distances reduce the saturation accordingly.

The APDs can successfully sense the signal level from the background, provided that a minimum event rate of 10 GHz can be delivered to its surface. Above this threshold, more background can be tolerated than with the SPAD detector. The exact level depends on the evaluation scheme used to extract timing information from the measurement result.

Results for a PDE of 20 % are given as a technologically reachable perspective but results for 5 % and 0.5 % are also presented because those systems are currently available on the market.

The APD in Figure 3a shows higher background tolerance than the SPAD detector. It scales well with higher laser event rates and can successfully evaluate scenarios in which the laser event rate is orders of magnitude lower than the background rate. This is especially distinct because of the high number of accumulated laser shots N.

The SPAD detector in Figure 3b requires lower laser power than the APD. The scenario is generally challenging for the SPAD detector because it operates in single-photon acquisition mode and is hence subject to saturation, further emphasized by the target distance of 100 m.

Dashed and dashed/dotted lines show system behavior for lower values of the PDE $\eta_{\mathrm{PDE}}$ with the highest of the three values of $\eta_{\mathrm{PDE}}=0.2$ derived from SPAD with enhanced sensitivity in the near infrared in [29]. With higher PDE, a lower background event rate can be tolerated than in the low PDE case, because the finite detection efficiency prevents saturation through incident sunlight. Thus, the detector requires a lower signal level for sensing but can also tolerate lower incident background radiation. This effect can be mitigated by using a smaller receiver aperture, enabling more compact system designs.

As SPAD-based detection schemes are a rather novel development, improvement in evaluation schemes is to be expected. While we defined an SNR level of 3 as an evaluable but challenging threshold, we expect that the minimum SNR level of SPADs can be further improved by temporally and spatially adaptive processing of different SPAD groups, see [30,31].

**Figure 4:**
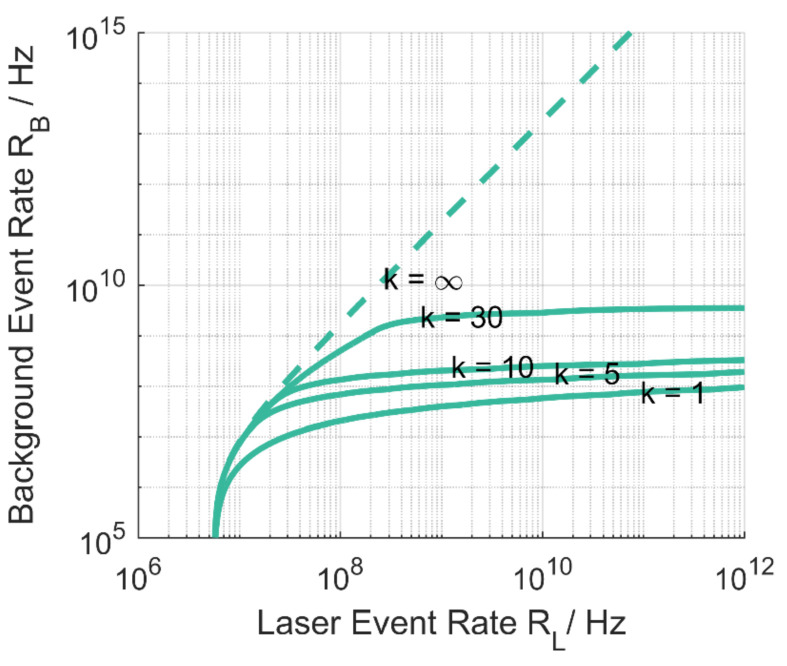
Behavior of the SPAD detector for higher multi-event levels k and limiting case of the linear detector. $N_{\mathrm{Ph}}$ is set to 30 following from dead time and system range. SNR = 3 contour drawn for $\eta_{\mathrm{PDE}}=0.2$, multi-event levels k and the linear detector (dashed line). Line for k = 1 is equivalent to Figure 3b.

Better saturation mitigation from multi-event using finite photon detection level or free-running mode with photon acquisitions to the maximum number of detections $N_{\mathrm{Ph}}$ (Equation (27)) are shown in Figure 4 for the SPAD detectors. The graph for k=∞ is the limit for a linear detector with $\eta_{\mathrm{PDE}}=0.2$ which is only reachable asymptotically by a detector without dead time.

The acquisition modes do not change the minimum required laser event rate on the detector for successful detection but show their advantage in scenarios with increasing background level.

Different research groups have picked up the task of improving SPAD ranging performance. Usage of coincidence detection [32] makes it possible to achieve higher background light immunity. On-chip integration of coincidence circuits has been demonstrated [33]. The selective reduction in incident event rates can enable successful detection, provided high enough laser power can be delivered to the detector’s surface.

The use of gating schemes [30] can further improve SNR values by preventing detector saturation before the pulse arrival if scene information and sufficient measurement time is available. Due to the SPADs’ digital behavior, the measurement procedure and data processing scheme can be adaptively changed if environmental conditions as fog, rain or snow influence the performance.

Combining those measures enables the SPAD to reach sufficient SNR for lower values of $r_{L}$ and higher background light levels $r_{B}$, but only partially bridging the gap to the higher APD background light level tolerance in high signal power scenarios.

### 3.2. Multi Pixel Detectors

The difference in common detector diameters suggests a comparison in which the SPAD takes up less space than the APD. When using the same optical system, this would lead to a reduction in power on the surface of the individual detector. The good array capabilities of SPADs suggest a multi pixel detector as a feasible alternative for comparison.

**Figure 5:**
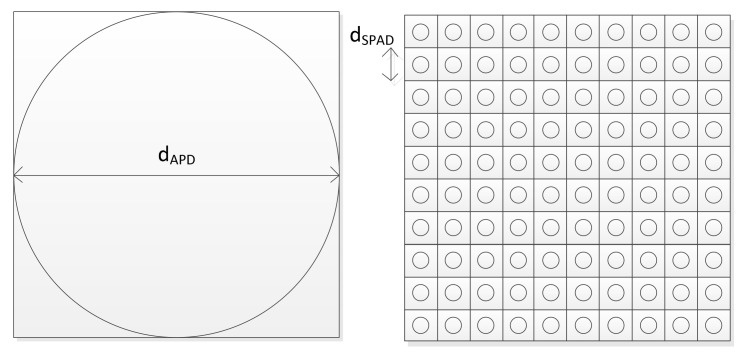
Comparison case for a single pixel APD detector of diameter 500 µm and a SPAD detector with 100 pixels and respective diameter of 50 µm.

Detector geometry for the comparison scenarios between APD and SPAD is shown in Figure 5. A single pixel APD detector with a diameter of 500 µm is implemented in an optical range finder scenario. To be able to compare the different detectors, a square illumination on the detector’s surface is selected.

Equation (30) with input rates multiplied by finite detection efficiency of SPAD and (14) for the APD case are used. To create a comparison scenario, the respective APD geometry is placed in the illuminated spot. The geometry results in an effective reduction in fill-factor. For the 500 µm diameter APDs, a value of $\eta_{\mathrm{FF},500}=\frac{\pi }{4}=78.5\;\%$ is achieved. 

Assuming a pixel pitch of 50 µm for the SPAD, 100 SPAD devices can be implemented in the same detector area as one 500 µm APD. Using commonly evaluated multiple pixels has implications for total system performance. By splitting up event rates between multiple pixels, absolute values of rate per pixel are reduced. Noise characteristics which are independent of signal level stay constant for both APDs and SPADs and thus define the array behavior for high numbers of pixels. It is generally easier to implement high numbers of SPADs in an array because of their purely digital evaluation scheme.

**Figure 6:**
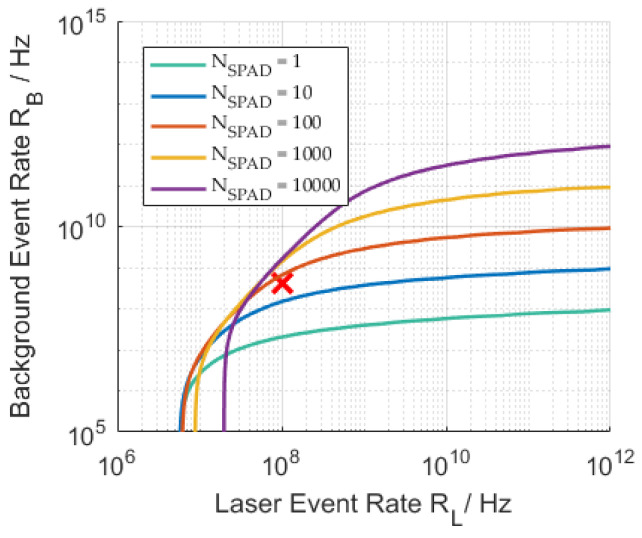
SNR = 3 contours of the multi pixel SPAD detector with $\eta_{\mathrm{PDE}}=0.2$, k=1 for different pixel numbers $N_{\mathrm{SPAD}}=\left[1,\;10,\;100,\;1000,\;10,000\right]$ plotted over total event rates $R_{B}$ and $R_{L}$. Other values from Table 2. Red cross: Multi pixel scenario used in Figure 7.

Multi pixel SPAD detectors reduce the absolute background event rate compared to the single pixel detector as seen in Equation (30) and Figure 6, resulting in a lower value of saturation $P_{A,M}$ up until the time-of-flight $t_{\mathrm{ToF}}$.

This is especially visible in the case of single-photon acquisition but also effects higher order photon detection, as seen in the figure. One issue is that the size of timestamp in bit generated by a SPAD depends on the maximum acquisition range $t_{\mathrm{ToF}}$ and bin size $t_{\mathrm{Bin}}$ which means that the amount of data produced by the array is also increased by factor $N_{\mathrm{SPAD}}$, creating a challenge for readout design.

Splitting incident photon rates onto a SPAD array can have benefits or draw backs depending mainly on the dark count rate. It constitutes the lower noise limit per pixel.

**Figure 7:**
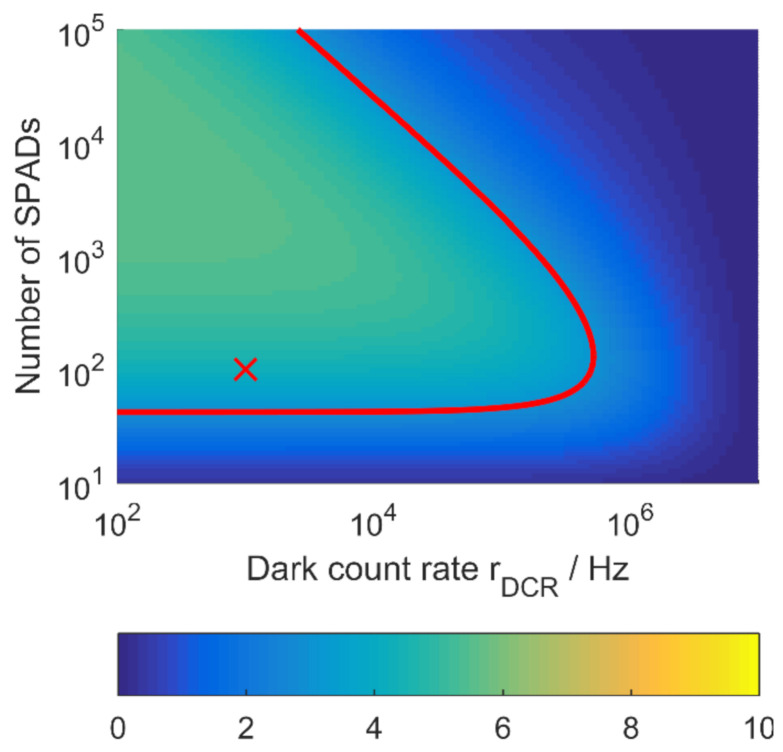
SNR from (30) for constant event rates $r_{L}=0.1\;\mathrm{GHz}$, $r_{B}=0.4\;\mathrm{GHz}$. SPAD and scenario parameters from Table 2 and Table 3 respectively. Red contour for SNR = 3. Red cross: multi pixel SPAD with $N_{\mathrm{SPAD}}=100$ and $r_{\mathrm{DCR}}=1\;\mathrm{kHz}$.

The usage of more SPADs can, furthermore, lead to the deterioration of system performance as shown in Figure 7. The fixed noise of a single pixel is multiplied by the number of pixels as seen in (30). There is an optimum to be found here between background light suppression, quantity of timestamp data and present dark count rate. Generally, smaller SPADs also exhibit lower DCR. The plot also shows that this particular event rate scenario (marked with a red X) is not successfully evaluable with a single SPAD in single-photon acquisition mode. The SPAD based on parameters in Table 2 shows in this example that increasing the SPAD number up to 1000 would still be beneficial, due to the low DCR used here. It has to be kept in mind, though, that the DCR will increase with rising temperatures.

**Figure 8:**
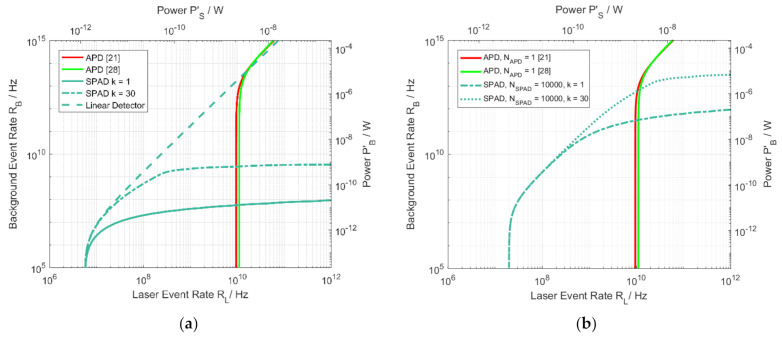
Contour lines for receiver performance when comparing a single APD to an equally sized SPAD detector (**a**) in different operation modes of the SPAD (single/multi-event detection) and (**b**) additionally for 10,000 SPAD pixels $N_{\mathrm{SPAD}}$. Contour lines according to (14) and (30) with values from Table 1, Table 2 and Table 3 at SNR = 3 are shown in each case.

The SNR comparison for a single 500 µm APD and a single SPAD with the same impinging number of photons per second on the detectors surface with scenario parameters from Table 3 is shown in Figure 8a. As before, the SPAD requires lower laser power than the APD for successful detection. As soon as this power threshold is reached, the APD will show a more background-tolerant behavior.

The SNR comparison for a single 500 µm APD and 100 SPADs is shown in Figure 8b. Although one cannot assume that both diode’s signals are evaluable at exactly the same SNR, the contour line of SNR 3 is again drawn for purpose of demonstration. It can be seen that higher background event rates are tolerable with the multi pixel SPAD array compared to the single pixel SPAD detector. On the other hand, the minimum required laser power is slightly higher as well. To show this effect more clearly, a relatively high number of SPADs of $N_{\mathrm{SPAD}}=10,000$ was chosen for this particular comparison. The advantage from multi-event acquisition shifts to higher laser event rates because of the overall reduced rates per pixel. The behavior of the APD is the same as in Figure 3 except for the slight reduction in fill-factor. As in the single pixel comparison, it requires higher laser event rates but can also tolerate more background photons.

When the detectors are not evaluated together but on a per pixel basis, the multi pixel SPAD system can achieve inherent spatial resolution. In LiDAR systems this translates into a smaller field-of-view which is also called the instantaneous Field-of-View (iFoV). The smaller reception angles lead to lower background light level for smaller detectors and thus lower background light influence. The limited array capability of APDs caused by process-based fluctuations in breakdown and biasing voltages and their analog circuitry causes difficulties in achieving the highest possible spatial resolution, requiring beam-steering solutions. The possibility for accumulating the same scene is limited for those scanning systems because of limited beam-steering velocity through rotating macroscopic or MEMS mirrors.

For SPADs, those process-based fluctuations exist as well but do not impact performance as drastically. Different breakdown and biasing voltages will lead to minimal and for most applications neglectable changes in timing precision for the diodes but their influence on charge multiplication does not affect system performance in the same way it does for APDs. The avalanche breakdown’s saturating behavior makes multiple detectors behave more uniformly.

## 4. Discussion and Outlook

This work analyses optical detection systems built from APDs or SPADs based on a common figure of merit, the signal-to-noise ratio. Besides their respective detectors noise behavior and signal detection mechanisms, we discuss currently achievable technologically determined parameters like fill-factor and respective detector sensitivity. 

The APD is more robust against incident background light radiation but requires higher signal power levels for detection. Its limited capability for 2D-array integration means it requires a beam-steering solution for achieving spatial resolution when used for a LiDAR system. APDs are a rather mature technology and have been well-optimized, making technological progress harder to achieve and thus more expensive.

SPADs show their advantages especially in low-power scenarios. Their smaller size compared to APDs generally results in both lower incident background light and signal levels, and their small depletion layer results in high temporal resolution. If background light levels saturate the SPADs, acquisition of multiple photon arrivals can overcome noise levels without introducing any new detector noise. Combination with coincidence detection logic can help achieve the highest possible ranges.

An increase in fill-factor and thus PDE is a promising direction for further development. Fill-factor improvements for SPAD arrays can stem from the use of integrated microlenses, but they also reduce the acceptance angle of the array, limiting their effect. The integration of readout circuitry on a separate wafer and subsequent bonding of readout and wafer with integrated backside-illuminated SPADs (BSI) promise high fill-factor designs and are ongoing areas of research. Low-noise and sensitive SPADs optimized for detection in the near infrared in a CMOS compatible process can be combined with high density read-out electronics in a smaller technology node.

Higher density SPAD integration also allows for an inherent spatial resolution. Especially for flash LiDAR systems which acquire scene depth information without the use of moving parts, this higher number of detectors can provide scene-dependent flexibility. A tradeoff can be found between high spatial resolution by evaluating all detectors by themselves and high range by commonly building histograms over bigger regions of the sensor. Algorithmic advances balancing the amount of data from the SPAD array which in multi-event detectors is activity based and detector ranging performance, can help to find the right operational conditions at the right time, narrowing the gap between APD and SPAD detectors for high distance ranging applications.

In summary, the SNR comparison of the two different photodetectors and their characteristics must be continued in further research. The analytical values of SNR would have to be coupled with object detection and image processing results to achieve a more detailled comparison. Our results lay the groundwork for further and more detailled discussions on the commonalities of the two approaches. The aim is to unite both worlds of photodiodes in order to develop the optimal LiDAR system of the future.

## Figures and Tables

**Table 1:** APD Parameters.

Parameter	Symbol	Unit	[21]	[28]	[7]
Dark Current	$I_{d}$	nA	0.8	0.5	0.1
Excess Noise Factor	F	-	2.5	-	13
Diameter	$d_{\mathrm{APD}}$	µm	500	500	500
Responsivity ^1^	R	A/W	58	50	40
Quantum Efficiency ^1^	$\eta_{\mathrm{QE}}$	-	0.8	0.85	-
Active Area	$A_{\mathrm{Opt}}$	mm^2^	0.196	0.25	0.25
Rise Time	$t_{r}$	ns	0.55	0.45	-
Amplification	M	-	100	100	100
Number of APDs	$N_{\mathrm{APD}}$	-	1	1	1

^1^ For radiation with wavelength λ=905 nm.

**Table 2:** SPAD parameters.

Parameter	Symbol	Unit	Value
Dark Count Rate	$r_{\mathrm{DCR}}$	Hz	1000
Afterpulsing	$P_{\mathrm{AP}}$	-	<1%
Dead Time	$t_{D}$	ns	20
Bin Width	$t_{\mathrm{Bin}}$	ns	8
Pixel Pitch ^1^	$d_{\mathrm{SPAD}}$	µm	50
Number of SPADs ^1^	$N_{\mathrm{SPAD}}$	-	100 $N_{\mathrm{APD}}$

^1^ For multi pixel detector comparison.

**Table 3:** Common parameters for ranging scenario.

Parameter	Symbol	Unit	Value
Time-of-Flight	$t_{\mathrm{ToF}}$	ns	667
Object distance	$d_{\mathrm{Max}}$	m	100
Pulse Duration	$t_{P}$	ns	8
Wavelength	λ	nm	905
Number of Laser shots	N	-	1000

## Data Availability

Not applicable.
